# Cumplimiento de la lista de verificación de seguridad de la cirugía en un hospital de Santander. Un estudio de corte trasversal

**DOI:** 10.15649/cuidarte.2122

**Published:** 2021-10-06

**Authors:** Martha Cecilia Sepúlveda Plata, Luis Alberto López Romero, Sandra Beatriz González

**Affiliations:** 1 Hospital Regional Manuela Beltrán. Fundación Universitaria de San Gil. Grupo de investigación en ciencias de la educación y de la salud, San Gil (Santander Colombia). E-mail:marthacirugia@hotmail.com Autor de correspondencia Fundación Universitaria de San Gil Fundación Universitaria de San Gil Colombia marthacirugia@hotmail.com; 2 Fundación Cardiovascular de Colombia. Grupo de Investigación y Desarrollo de Conocimiento en Enfermería FCV (GIDCEN- FCV). Centro de investigaciones, Floridablanca (Santander, Colombia). E-mail: alberlop25@hotmail.com Fundación Cardiovascular de Colombia Colombia alberlop25@hotmail.com; 3 Instituto Mexicano del Seguro Social, División de Programas de Enfermería (Ciudad de México, México). Catedrática de posgrado en Educación a Distancia UNIVERSIDAD CUAUHTÉMOC. Plantel Aguascalientes, México. E-mail: sandrabeatrizmota@gmail.com Instituto Mexicano del Seguro Social Instituto Mexicano del Seguro Social Mexico

**Keywords:** Cumplimiento, Calidad de la Atención de Salud, Lista de Verificación, Incidentes, Cirugía, Seguridad del Paciente., Compliance, Quality of Health Care, checklist, Incidents, Surgery, Patient Safety., Cooperação, Qualidade da Assistência à Saúde, Lista de Checagem, Incidentes, Cirurgia, Segurança do Paciente.

## Abstract

**Introducción:**

La seguridad del paciente constituye una prioridad en la atención en salud, siendo la lista de verificación para la seguridad quirúrgica una de las estrategias implementadas por la OMS. El objetivo fue determinar el nivel de cumplimiento en la aplicación de la lista de verificación de seguridad de la cirugía en personal de sala quirúrgica de una institución pública.

**Materiales y Métodos:**

Estudio de corte transversal en 45 miembros del equipo quirúrgico de un hospital, en los cuales se evaluó el cumplimiento a la lista de chequeo de la OMS durante el mes de julio y agosto del año 2018.

**Resultados:**

El cumplimiento global fue del 13.3% (n=6), siendo la fase previa a la anestesia la que alcanzó el mayor nivel (55.6%, n=25). El mayor cumplimiento lo registró el personal de instrumentación quirúrgica (100%, n=8), mientras el más bajo el personal de enfermería (25%, n=3), con diferencias estadísticamente significativas (p=0.005). Adicionalmente, se observó una correlación entre los años de trabajo en el servicio y el cumplimiento en fase de transferencia (rho= -0.30, p=0.048).

**Discusión:**

El cumplimiento general fue bajo, lo cual corrobora la hipótesis planteada y resulta similar a otros estudios descritos en la literatura.

**Conclusiones:**

El cumplimiento general a la lista de chequeo fue muy bajo, con comportamientos diferenciales al ser las instrumentadoras quirúrgicas las que presentaron mayor cumplimiento y el personal de enfermería el menor. El ítem de profilaxis antibiótica el de menor cumplimiento, mientras que la fase previa a la anestesia la de mayor nivel.

## Abstract (en)

**Introduction:**

Patient safety constitutes a priority in health care, being the surgical safety checklist one of the strategies implemented by the WHO.

**The objective:**

was to determine the level of compliance in the application of the surgical safety checklist in surgical room personnel of a public institution.

**Materials and Methods:**

Cross-sectional study in 45 members of the surgical team in a hospital, in which compliance to the WHO checklist was evaluated during July and August 2018.

**Results:**

Overall compliance was 13.3% (n=6), with the pre-anesthesia phase reaching the highest level (55.6%, n=25). The highest compliance was recorded by the surgical instrumentation staff (100%, n=8), while the lowest by the nursing staff (25%, n=3), with statistically significant differences (p=0.005). Additionally, a correlation was observed between years of work in the service and compliance in the transfer phase (rho= -0.30, p=0.048).

**Discussion:**

Overall compliance was low, which corroborates the stated hypothesis and is similar to other studies described in the literature.

**Conclusions:**

The overall compliance to the checklist was very low, with differential behaviors as the surgical instrument technicians showed the highest compliance and the nursing staff the lowest. The antibiotic prophylaxis item was the least compliant, while the pre-anesthesia phase was the most compliant.

## Resumo

**Introdução:**

A segurança do paciente é prioridade na assistência à saúde, sendo o checklist de segurança cirúrgica uma das estratégias implantadas pela OMS. O objetivo foi determinar o nível de conformidade na aplicação da lista de verificação de segurança cirúrgica na equipe de centro cirúrgico de uma instituição pública

**Materiais e métodos:**

Estudo transversal em 45 membros da equipe cirúrgica de um hospital, no qual foi avaliada a conformidade com o checklist da OMS durante os meses de julho e agosto de 2018

**Resultados:**

A adesão geral foi de 13,3%(n=6), sendo a fase anterior à anestesia a que atingiu o nível mais alto (55,6%, n=25). A maior conformidade foi registrada pela equipe de instrumentação cirúrgica (100%, n=8), enquanto a menor foi pela equipe de enfermagem (25%, n=3), com diferenças estatisticamente significativas (p=0,005). Além disso, foi observada correlação entre anos de trabalho no serviço e adesão na fase de transferência (rho = -0,30, p=0,048).

**Discussão:**

A adesão geral foi baixa, o que corrobora a hipótese levantada e é semelhante a outros estudos descritos na literatura.

**Conclusões:**

O cumprimento geral do checklist foi muito baixo, com comportamentos diferenciados, visto que a instrumentação cirúrgica foi a que mais aderiu e a equipe de enfermagem menos. A profilaxia antibiótica item a de menor complacência, enquanto a fase pré-anestésica a de maior grau.

## Introducción

Según la Organización Mundial de la Salud (OMS) los eventos adversos por falta de atención segura constituyen un grave problema de salud pública, siendo una de las 10 principales causas de muerte y discapacidad en el mundo, estimando 134 millones de eventos adversos por año en los hospitales de los países de ingresos bajos y medios, de los cuales 2,6 millones resultan mortales (1).

Datos de la OMS muestran que al año a nivel mundial se llevan a cabo alrededor de 234 millones de intervenciones de cirugía mayor. Adicionalmente, se sabe que las complicaciones atribuibles a intervenciones quirúrgicas causan discapacidades o prolongan la estancia hospitalaria entre un 3% y un 25% de los pacientes; es decir 7 millones de pacientes al año están a riesgo de complicaciones posteriores a la cirugía. Así mismo, se conoce que por los menos un millón muere cada año luego de someterse a una cirugía; datos que permiten estimar que los eventos adversos derivados de estos constituyen un problema serio de salud pública global (2).

En el año 2004 la OMS decretó la creación de la Alianza Mundial por la Seguridad del Paciente y adicionalmente precisaron las pautas para reducir los errores en la atención en salud. En 2008 este mismo organismo suma a dicha alianza un reto adicional de seguridad a través de la campaña denominada «Cirugía segura salva vidas», con el propósito de optimizar la seguridad de las cirugías a nivel global a través del establecimiento de un conjunto de indicadores transversales y globales denominado, lista de verificación quirúrgica. Esta lista está compuesta por 19 ítems que se deben chequear en todo el proceso quirúrgico: antes, durante y después de la intervención (2), (3).

Por otra parte, en el estudio ENEAS (Estudio Nacional sobre los Efectos Adversos Ligados a la Hospitalización) realizado en España, se encontró un porcentaje similar de eventos adversos entre los servicios médicos (8,86%, IC 95%: 7,73-10,0) y quirúrgicos (8,07%, IC 95%:7,12-9,01), mientras que la incidencia en los servicios de cirugía general fue del 10,5%, y del 3% en los servicios de cirugía mayor ambulatoria, siendo 36% de estos evitables (4).

En el estudio IBEAS: Prevalencia De Efectos Adversos En Hospitales De Latinoamérica, llevado a cabo en 5 países (Argentina, Colombia, Costa Rica, México y Perú) y 58 centros hospitalarios, se encontró que la prevalencia de Eventos Adversos fue del 10.5% (n=1.191 pacientes). Adicionalmente, se identificó una incidencia global de 19.8%, siendo alrededor de un 60% de los Efectos Adversos (EA) considerados evitables. En el caso particular de Colombia se sabe que la prevalencia de EA fue del 7.7% (IC 95%:6.8-8.7), es decir 7 de cada 100 pacientes ingresados a una institución de salud podrían sufrir algún daño atribuido a la atención. Así mismo, se estimó una incidencia acumulado del 12,5% y adicionalmente que el 62,2% de los EA eran evitables (5).

Gonzales y colaboradores en un estudio realizado en Cali, Colombia en 2015 con el objetivo de determinar las causas que ocasionan la presencia de sucesos adversos relacionados con el acto quirúrgico en una institución de salud de tercer nivel, encontraron que el 58,44 de los suceso adversos se clasificaron como prevenibles, 13,64% como eventos adversos no prevenibles, 22,73% como acciones inseguras y el 5,19% como incidentes. Las especialidades médicas donde surgieron los EA correspondieron en su mayoría a cirugía general con 28,10%, seguida de anestesiología con 18,30% y en tercer lugar ortopedia 13,07% (6). Por su parte, Batista y colaboradores (7) realizaron en 2019 un estudio en Brasil con el propósito de estimar la prevalencia y evitación de eventos adversos quirúrgicos en el hospital; en dicho estudio se encontró una prevalencia de eventos adversos quirúrgicos del 21,8%. Las fallas técnicas quirúrgicas contribuyeron en aproximadamente el 40% de los casos. Se destacan los eventos adversos relacionados a la infección del sitio quirúrgico (30%, n=18), la dehiscencia de sutura quirúrgica (16,7%, n=10) y el hematoma/seroma (15%, n=9).

Además, la lista de verificación de seguridad quirúrgica ha sido implementada en diferentes países del mundo con resultados variables. White y colaboradores (8) en una revisión sistemática y metanálisis de 47 estudios realizada en 2021 con el objetivo de identificar las estrategias de implementación utilizadas en la aceptación de la lista de verificación de seguridad quirúrgica de la OMS en los Países De Ingresos Bajos y Medios (PIBM); examinar cualquier asociación de estrategias de implementación con la efectividad de la implementación; y evaluar el impacto clínico. En dicho estudio se encontró que el uso de la lista de verificación de seguridad quirúrgica se asoció con una reducción de la mortalidad (RR 0,77; IC del 95%: 0,67 a 0,89), de todas las complicaciones (RR 0,56; IC del 95%: 0,45 a 0,71) y complicaciones infecciosas (RR 0,44; IC del 95%: 0,37 a 0,52).

En síntesis, los eventos adversos son un problema de salud pública tanto a nivel mundial, de Latinoamérica y Colombia. Adicionalmente, se sabe que aproximadamente la mitad de dichos eventos pueden ser debidos a procedimientos quirúrgicos y en general sean eventos quirúrgicos o no, la mitad e incluso más podría ser evitables implementado barreras de seguridad del paciente.

Teniendo en cuenta todo lo anterior la hipótesis del presente estudio fue que el nivel de cumplimiento por parte del personal de cirugía en la aplicación de la lista de chequeo era inferior al 60% en la institución pública del departamento de Santander, Colombia. Dado todo el contexto anterior, el objetivo del presente estudio fue determinar el nivel de cumplimiento en la aplicación de la lista de verificación de seguridad de la cirugía a través del diligenciamiento de la lista de chequeo de la OMS, para así evaluar el nivel de adherencia del personal al instrumento.

## Materiales y Métodos

Diseño y población de estudio: Estudio de corte transversal, llevado a cabo en el personal del servicio de cirugía de una institución pública del departamento de Santander, Colombia, el cual estaba conformado por 45 personas (24 médicos especialistas, 12 auxiliares de enfermería, un enfermero jefe y 8 instrumentadores quirúrgicos).

Instrumento de recolección y aplicación de la lista: Se adaptó a la institución el instrumento “*lista de verificación de la seguridad en la cirugía”*, creado por la OMS en el año 2008 (3). Dicha lista es un instrumento válido, confiable y estandarizado. La lista evalúa en total 42 ítems, distribuidos en tres momentos; 20 ítems correspondientes a la fase de transferencia; el segundo momento es previo a la anestesia con 15 ítems; el tercer momento corresponde a previo traslado a URPA (Unidad de Recuperación Pos Anestésica) con 7 ítems; para un total de 42 ítems. Cada uno de los ítems de dicha lista corresponde a una variable del instrumento en el cual la totalidad son de naturaleza cualitativa y son medidas en escala nominal. La técnica que se empleó para la aplicación del instrumento fue la de observación para corroborar el cumplimiento correcto de la lista de verificación de la seguridad en cirugía por parte del personal quirúrgico que conforma el servicio de cirugía de la institución.

La evaluación del cumplimiento de cada una de las variables de la lista se realizó de la siguiente manera. Una variable fue considera como que “si cumplía” si la persona encargada de liderar el proceso de diligenciamiento de la lista formula de manera correcta los interrogantes e interactuaba adecuadamente con el paciente y los demás miembros del equipo quirúrgico, confirmando que el equipo había llevado a cabo todas las actividades, para posteriormente diligenciar las casillas pertinentes de la lista. Mientras que una variable fue considerada como “no cumple” cuando los miembros del equipo omitían la lista de verificación o algunos de sus interrogantes y proseguían con la siguiente fase del acto quirúrgico sin haber confirmado que el equipo había realizado a cabalidad sus respectivas actividades.

Adicionalmente a la aplicación del anterior instrumento se indagaron por aparte entre los miembros del equipo quirúrgico algunas variables sociodemográficas como la profesión, el sexo, los años de experiencia profesional y el tiempo que llevaba trabajando en el servicio. Las variables profesión y sexo que son de naturaleza cualitativa fueron medidas en escala nominal, mientras que las variables años de experiencia profesional y el tiempo que lleva trabajando en el servicio que son variables cuantitativas fueron medidas en escala de razón.

Análisis estadístico: La información fue doblemente digitada y validada en dos hojas de cálculo de Microsoft Excel. Las variables cualitativas fueron descritas con frecuencias absolutas y relativas, mientras que las variables cuantitativas fueron descritas como promedios acompañados de su desviación estándar, dado que presentaron una distribución normal según la prueba de Shapiro Wilk, la prueba Kurtosis y demás pruebas gráficas (histogramas, diagramas de cajas y gráficas como QQ plot). Adicionalmente, un análisis bivariado fue llevado a cabo empleado una prueba exacta de Fisher o JI cuadrado con la finalidad de evaluar las diferencias entre los niveles de cumplimiento y las variables de interés (tipo de personal, sexo, tiempo de experiencia profesional y tiempo de trabajo en servicio). Así mismo, se realizó un análisis de correlación de Pearson entre los niveles de cumplimiento general y por las diferentes fases de la cirugía (transferencia, preanestesia y previo URPA) y el cumplimiento por profesional, y los años de experiencia laboral y los años de trabajo servicio. Se consideró como significativo todos los valores *p* inferior a 0,05. Todas las pruebas de hipótesis fueron realizadas a dos colas y los datos fueron analizados empleando el paquete estadístico STATA, versión 14.0.

Consideraciones éticas: Esta investigación fue catalogada sin riesgo, según el artículo 11 de la resolución 8430 (9), dado que solo se emplearon técnicas de entrevista, cuestionarios y observación directa del comportamiento de cumplimiento de los miembros del equipo quirúrgico. Los miembros del equipo quirúrgico firmaron el consentimiento informado por escrito. Adicionalmente, el presente estudio fue aprobado por el Comité de Ética de la Institución Prestadores de Servicios de Salud (IPS) en donde se desarrolló el estudio mediante el Acta No.2 de febrero 28 de 2018 y cumplió con la normatividad nacional e internacional para la investigación en seres humanos (9), (10).

## Resultados

Un total 45 miembros del equipo quirúrgico e igual número de listas fueron evaluados, los cuales en su mayoría eran hombres (57.8%), el promedio de edad fue un 41.8± DE 12.0 años y el promedio de años de experiencia profesional fue de 18.3 ± 11.7 años, Tabla 1.

**Tabla 1:** Características sociodemográficas de la muestra

Característica n	N (%) o Promedio ± DE
Sexo		
Hombre	26(57.8)
Mujer	19(42.2)
Edad, años	41.84± 12.0
Tipo de Personal		
Auxiliar enfermería	12(26.7)
Anestesiólogos	4(8.9)
Instrumentadoras	8(17.8)
Medico	20(44.4)
Enfermero	1(2.2)
Años de experiencia laboral	18.3±11.7
Años de trabajo en servicio	6.0±3.9

Fuente: Elaboración propia

En la Tabla 2 se puede observar que el cumplimiento general de las listas fue del 13.3%, mientras que el momento de la cirugía previo a la anestesia alcanzó un 55.6%, seguido del momento previo a URPA que registro un cumplimiento del 37.78% y finalmente la fase de transferencia con un 24.44%.

**Tabla 2:** Cumplimiento global y por fases del acto quirúrgico

Número de ítem cumplido por el personal	n (%)
Cumplimiento global (42 ítems=cumplimiento total)		
42 ítems	6(13.33)
41	2 (4.44)
40	5(11.11)
39	6(13.33)
38	9 (20.00)
37	3(6.67)
36	4(8.89)
35	3(6.67)
34	4(8.89)
32	2(4.44)
31	1(2.22)
Cumplimiento previo a URPA (7 ítems=cumplimiento total)		
7	17(37.78)
6	16(35.56)
5	9(20.00)
4	2(4.44)
3	1(2.22)
Cumplimiento previo a la anestesia (15 ítems=cumplimiento total)		
15	25(55.6)
14	9(20.00)
13	1(2.22)
11	4(8.89)
10	3(6.67)
9	1(2.22)
8	2(4.44)
Cumplimiento transfer, ítem total (20 ítems=cumplimiento total)		
20	11(24.44)
19	11(24.44)
18	7(15.56)
17	10(22.22)
16	3(6.67)
15	2(4.44)
14	1(2.22)

Fuente: Elaboración propia

**Figura 1:**
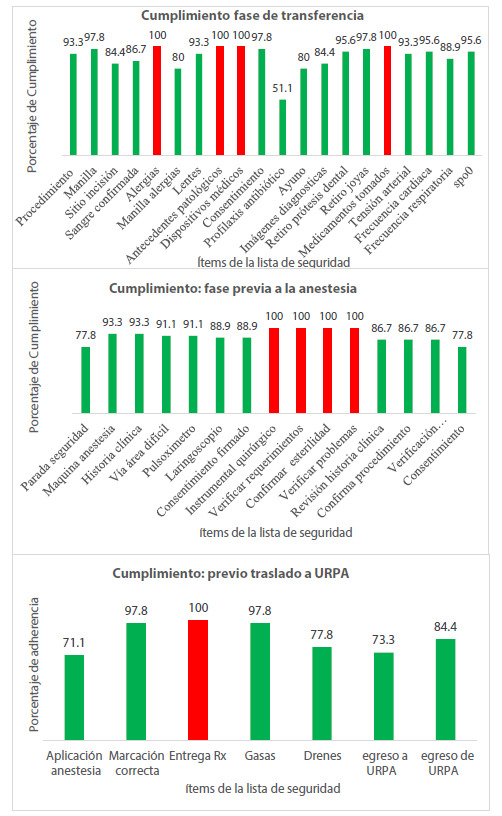
Cumplimiento por fases el acto quirúrgico

**Tabla 3:** Análisis de niveles de cumplimiento profesión por variables de interés

Características	Cumplimiento total			Cumplimiento transferencia			Cumplimiento Preanestesia			Cumplimiento previo URPA		
	Si	No	P valor	Si	No	P valor	Si	No	P valor	Si	No	P valor
Sexo			0.636*	7(63.6)	19(55.9)	0.651*	13(52.0)	13(65.0)	0.380*	12(70.6)	14(50.0)	0.175*
Hombre	4(66.7)	22(56.4)										
Mujer	3(23.3)	17(43.6)		4(36.4)	15(44.1)		12(48.0)	7(35.0)		5(29.4)	14(50.0)	
Edad												
< 20 años	5(83.3)	20(51.3)	0.141*	8(72.7)	17(50.0)	0.187*	14(56.0)	11(55.0)	0.947*	8(47.06)	17(60.7)	0.371*
>21 años	1(16.7)	19(48.7)		3(27.3)	17(50.0)		11(44.0)	9(45.0)		9(52.94)	11(39.3)	
Años de servicio												
< 5 años	5(83.3)	21(53.9)	0.173*	8(72.7)	18(52.9)	0.248*	16(64.0)	10(50.0)	0.345*	9(52.9)	17(60.7)	0.609*
> 5años	1(16.7)	18(46.2)		3(27.3)	16(47.1)		9(36.0)	10(50.0)		8(47.1)	11(39.3)	
Profesión												
Aux Enfermeria	3(25.0)	9(75.0)	0.005**	3(25.0)	9(75.0)	0.747**	8(66.67)	(4) 33.33	0.517**	5(41.67)	7(58.33)	0.894**
Enfermeras	0(0.0)	1(0.0)		0(0.0)	1(0.0)		0(0.00)	1(100.00)		0(0.00)	1(100.00)	
Instrumentadoras	8(100)	0(0.0)		2(25.00)	6(75.00)		5(62.50)	(3) 37.50		2(25.00)	6(75.00)	
Anestesiólogos	2(50.0)	2(50.0)		2(50.00)	2(50.00)		1(25.00)	(3) 75.00		2(50.00)	2(50.00)	
Cirujanos	11(55.0)	9(45.0)		4(20.00)	16(80.00)		11(55.00)	9(45.00)		8(40.00)	12(60.00)	

*Prueba de Ji cuadrado, **Prueba exacta de Fisher. Fuente: Elaboración propia

En la Figura 1, se observa que los ítems de la lista que presentaron cumplimiento (100%) fueron alergias, antecedentes patológicos, dispositivos médicos, toma de medicamentos, instrumental quirúrgico completo, verificación de requisitos, confirmación de esterilidad y verificación de problemas con el instrumental, entrega de rayos X; mientras que el menor cumplimiento se observó en los ítems de profilaxis antibiótico con un 51.1%, aplicación de analgesia tras operatoria con un 71.1%, egreso a URPA con un 73.3%, parada de seguridad pre anestésica con un 77.8%, consentimiento firmado con un 77.8% y finalmente postura de la manilla alérgica y ayuno con un 80% cada uno.

En relación al cumplimiento por profesión, se encontró que el personal de instrumentación quirúrgica fue el que presentó el mayor nivel con un 100%, seguido de los cirujanos con un 55%, los anestesiólogos con un 50%, y finalmente el personal de enfermería con un 25%, con diferencias estadísticas entre estos, p=0.005. Así mismo, se observa que al llevar a cabo las pruebas de hipótesis no se hallaron diferencias estadísticamente significativas entre los niveles de cumplimiento global y desglosado por fases del momento de la cirugía, y las diferencias variables de interés como el sexo del profesional, los años de experiencia profesional y años de trabajo en el servicio, Tabla 3.

**Figura 2:**
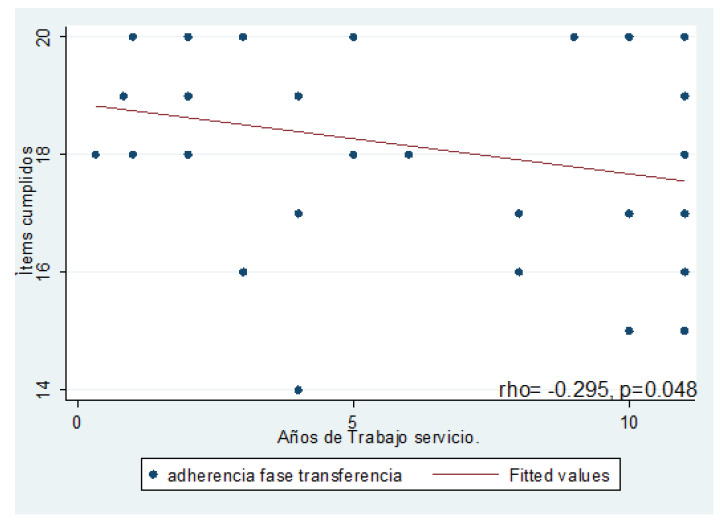
Correlación entre los años de trabajo en el servicio y el cumplimiento en fase de transferencia

Finalmente, no se encontró correlación entre los años de experiencia profesional y los años de trabajo en el servicio y el cumplimiento global, el cumplimiento profesional, el cumplimiento previo URPA, excepto en el caso del cumplimiento en la fase de transferencia y los años de trabajo servicio, en el cual se encontró un coeficiente de correlación de Pearson débil inverso (∫= -0.30, p=0.048), Figura 2.

Fuente: Elaboración propia.

## Discusión

En este estudio se evaluó la aplicación de la lista de chequeo de seguridad quirúrgica propuesto por la OMS a la totalidad del equipo quirúrgico del hospital; siendo en su mayoría hombres con amplio nivel de experiencia profesional. El cumplimiento general del equipo fue bajo y tuvo un comportamiento diferencial por fases del momento quirúrgico y por profesional del equipo; siendo el momento de la cirugía previo a la anestesia el de mayor cumplimiento y la fase de transferencia el más bajo; mientras que el personal de instrumentación quirúrgica el que presenta mayores niveles y el personal de enfermería el del nivel más bajo de cumplimiento. Además, se pudo evidenciar que el sexo del profesional, los años de ejercicio profesional y los años de trabajo en el servicio no hacen que este constructo tenga un comportamiento diferencial. Sin embargo, los años de ejercicio profesional se correlacionaron de manera inversa con el nivel de cumplimiento de la fase de transferencia.

La presente investigación constituye uno de los primeros estudios llevados a cabo en Colombia en donde se emplean un criterio estricto para evaluar el cumplimiento de los miembros del equipo quirúrgico (formula de manera correcta los interrogantes e interactuaba adecuadamente con el paciente y los demás miembros del equipo quirúrgico, confirmando que el equipo ha llevado a cabo todas las actividades y diligenciaba las casillas pertinentes de la lista) de una IPS a los ítems de la lista de seguridad quirúrgica propuesto por la OMS. Adicionalmente, en evaluar el cumplimiento entre los miembros del equipo, así como el cumplimiento por cada fase de la cirugía (transferencia, previo a la anestesia, previo a URPA) de manera individual.

En este estudio el cumplimiento general solo alcanzó un 13.3%, mientras que algunos ítems (alergias, antecedentes patológicos, dispositivos médicos, toma de medicamentos, instrumental quirúrgico completo, verificación de requisitos, confirmación de esterilidad, verificación de problemas con el instrumental, entrega de rayos x) evaluados de manera individual alcanzaron el 100% del cumplimento por parte del personal.

Se evidencio el diligenciamiento de la lista de verificación de seguridad de la cirugía y se observó a 45 integrantes del equipo quirúrgico, encontrándose en el personal auxiliar de enfermería falencias en el registro al dejar de marcar la casilla correspondiente al ítem de cada pregunta. Se evidenció con mayor frecuencia error en el diligenciamiento de los ítems de alergias ya que al interactuar con el paciente este refería no tener alergias, no obstante, se marcaba la casilla correspondiente a manilla indicando que, si era alérgico, por lo cual no hubo concordancia en este ítem.

También se observó confusión en las opciones de respuesta de la pregunta al no diferenciar la selección NO y NO APLICA, en algunas situaciones se diligenció el ítem sin interactuar con el paciente y en otras se interactuaba interrogándolo, pero no se registraba la marcación en la casilla, además se evidencia el diligenciamiento de la lista de manera operativa y mecánica por lo que no se le daba la importancia a la lista como herramienta para evitar eventos adversos, errores e incidentes. En el ítem de pregunta de profilaxis antibiótica, este se administraba, pero no hubo diligenciamiento de la hora de aplicación, igualmente sucedió con el ítem de ayuno, debido a que se interrogaba al paciente, pero el personal de enfermería no diligenciaba las horas de ayuno en la lista.

Otra falla evidenciada durante la fase previa a la anestesia, en el ítem parada de seguridad fue la falta de registro del responsable del diligenciamiento, debido a que, si se llevaba a cabo en presencia de todo el equipo quirúrgico, sin embargo, algunos especialistas no le daban la importancia como un instrumento útil para la seguridad del momento quirúrgico; presentado incomodidad y resistencia al formalizar el registro y comunicación con el paciente.

El auxiliar de enfermería se convierte en un personal clave para vigilar la ejecución de las acciones de cada integrante del equipo quirúrgico, por lo cual, aparte de ser el responsable del diligenciamiento e interacción de gran parte de la lista, debe continuamente motivar a todo el equipo a tener la conciencia de cumplir con cada ítem de verificación de esta.

En la fase previo traslado a URPA se encontró y se observó un inadecuado diligenciamiento en lo correspondiente a la aplicación de la analgesia, marcando la casilla, pero no registrando el medicamento, y en el ítem presencia de drenes no se hace claridad sobre cuales drenes. En el egreso de URPA se observó que se realizaba la lista de verificación de egreso y se entrega el instructivo de cuidados posoperatorios verificando el entendimiento por parte del usuario y su familia. De igual forma, en esta fase se evidencio el no cumplimiento de la identificación del responsable de quien da el egreso.

A pesar de que el proceso de interacción con el paciente y diligenciamiento de la lista no se cumplía en su totalidad el personal de enfermería es el líder y garante del seguimiento de las barreras de seguridad establecidos en la lista con un resultado exitoso. Además, cabe resaltar el compromiso del personal de enfermería con el diligenciamiento y cumplimiento, dado que se evidencio que en un número importante de ingresos de enfermos a salas de cirugía se ejecutaban las acciones seguras de manera efectiva.

En relación a lo observado y al diligenciamiento de los ítems correspondientes al médico anestesiólogo se evidencio la interacción con el paciente, realizando la verificación y cumplimiento, lo que lleva a determinar la importancia de mantener una comunicación efectiva con la persona objeto de cuidado y el personal responsable de la cirugía; resaltando la gran responsabilidad en la ejecución de las actividades por parte de este grupo de profesionales, sin embargo al momento de marcar la casillas no lo hacen, por lo cual la lista queda sin evidencia de la actividad y en consecuencia la calificación como no cumplimiento del ítem.

Por otro lado, se identificó y se observó que los profesionales de instrumentación quirúrgica ejecutaron todas las actividades correspondientes a los ítems, verificando e interactuando en su totalidad con el paciente y el equipo quirúrgico. Así mismo, los médicos cirujanos se evaluaron a través de la observación y verificación del registro de los ítems del instrumento, encontrando actitudes de rechazo, omisión de la firma, poca interacción con los pacientes, llenado mecánico, falta de consentimientos informados firmados, diligenciamiento de la lista en la fase no pertinente. De lo anterior se evidencia que falta mayor compromiso y responsabilidad en el uso de esta herramienta como estrategia para dar seguridad y reducir los posibles errores es incidentes evitables en el quirófano.

El bajo comportamiento de cumplimiento de los responsables del acto quirúrgico del hospital al diligenciamiento de la lista de chequeo podría ser explicado en parte por el criterio empleado para considerar como cumplimiento o no cumplimento a cada uno de los ítems; es decir en la presente investigación se tomó un criterio estricto de cumplimiento, el cual no incluía solo realizar la actividad por parte del personal, sino que además el registro dejando evidencia en la lista de verificación.

El bajo cumplimiento general a la lista de verificación encontrada en el presente estudio (13.3%), es concordante con varias investigaciones descritas en la literatura. En un estudio multicéntrico realizado en 9 hospitales de España, se evidencio un cumplimiento general del 27.8% (IC 95%:18.5- 37.0%) y porcentaje de cumplimiento de los ítems del 70.1% (IC 95%: 67.9%-72.2%) (11). Así mismo, en una investigación llevada a cabo en Brasil en el cual se revisaron 375 historias clínicas correspondientes a cirugías electivas, se encontró que el 61% tenían lista de verificación y que solo el 4% estaban completamente diligenciados (12). Así mismo, en un estudio realizado en el hospital universitario de São Pau, Brasil, se evaluaron 60 listas de chequeo correspondientes a pacientes pediátricos y se encontró que el no cumplimiento de la lista de chequeo alcanzó el 34.7% de los ítems (13); lo cual resulta similar a los hallazgos de este estudio (13.3%) con los reportados por la literatura en mención: 27.8% y 4% respectivamente.

Contrario al bajo cumplimiento general reportado en este estudio, el cual solo alcanzó el 13,3%, una investigación realizado por Dackiewicz y col (14), encontraron un cumplimiento del 85% luego de 18 meses de iniciado la implementación de la lista, sin embargo inicialmente en la prueba piloto el cumplimiento solo había alcanzado el 5%, para ir aumentado hasta el 48% en el tercer mes, y posteriormente un 65% a los 8 meses; situación que sugiere que el cumplimiento del personal aumenta conforme pasa el tiempo. Sin embargo, en este estudio dicho efecto no pudo ser visualizado, dado que se realizó una sola medición y además el hospital ya había adoptado como política de seguridad la aplicación de la lista de la OMS desde hace más de 2 años.

De igual forma, en un estudio realizado por Fourcade y col (15) en el cual evaluaron 1.440 procedimientos quirúrgicos y 1.299 listas de verificación de 18 centros de cáncer en Francia, los autores encontraron una tasa de cumplimiento del 90.2%, así mismo, lograron identificar 11 barreras para la implementación efectiva de las listas de chequeo, como lo fueron: la duplicación de elementos dentro de las listas de verificación existentes, la mala comunicación entre el cirujano y el anestesiólogo, el tiempo dedicado a completar la lista de verificación para ningún beneficio percibido y la falta de comprensión y calendario, ambigüedad, riesgos no contabilizados y una jerarquía tradicional. Estas barreras, aunque fueron percibidas como obstáculo que interfieran el diligenciamiento de la lista difieren un poco de las barreras percibidas en este estudio, dado que en el presente trabajo el bajo cumplimiento global podría obedecer a aplicación de un criterio más estricto para determinar el cumplimiento por parte del equipo.

Igualmente, en un estudio realizado, en México en el cual se evaluaron 326 cirugías, se encontró un cumplimiento global del 87.9%, así mismo, en este mismo estudio el 91.8% del personal considera que la lista es viable; para el 86.3%, proporciona algún beneficio, y el 91.2% consideraba que evita eventos adversos (16). También, contrario a este estudio en una investigación llevada a cabo en India con 300 listas pertenecientes a pacientes pediátricos, se encontró que el cumplimiento global alcanzo un 95.7% (17).

Así mismo, en un estudio realizado por Rodríguez (18), se encontró que el cumplimiento de todos los ítems por parte del personal superaba el 50%, lo cual es contrario a los hallazgos de este estudio, donde el cumplimiento global del personal solo alcanzó el 13.3%, sin embargo, el cumplimiento individual ítem a ítem es muy coincidente entre los dos estudios, dado que, en ambas investigaciones para la mayoría de estos, el cumplimiento superaba el 60%. En síntesis, contrario al cumplimiento estimando en este estudio que solo alcanzo el 13,3%, en los estudios en mención el cumplimiento general varía entre 50% al 95.7%.

En relación con el cumplimiento de manera individual para cada uno de los ítems de la lista de chequeo, en el presente estudio se encontró que los ítems de profilaxis antibiótico, aplicación de analgesia transoperatoria, egreso a URPA, parada de seguridad preanestésica, consentimiento firmado y finalmente postura de la manilla alérgica y ayuno, fueron los ítems que presentaron los niveles más bajos de cumplimiento por parte del personal del equipo quirúrgico. En un estudio realizado en México (16) a partir de la listas de verificación de la OMS adaptada para cirugías cardiacas; se encontró que los niveles de cumplimiento eran superiores a los encontrados en el presente estudio; los ítems con menor cumplimiento fueron: se ha completado la lista de verificación de circulación extracorpórea (19.2%) y marcaje del sitio quirúrgico (9.6%), durante la fase de antes de la inducción de anestesia; previsión de eventos críticos-cirujano (15.1%) y prevención de eventos críticos-anestesiólogo (35.6%), durante la fase de antes de la incisión cutánea; revisión de los principales aspectos de la recuperación (41.1%), el etiquetado de las muestras (28.8%), recuento completo de instrumental, gasas y agujas (21.9%), nombre del procedimiento (19.2%), firma cirujano (12.3%), firma anestesiólogo (9.6%) y ocurrencia de eventos adversos (9.6%) para la fase de previo a la salida del quirófano.

Igualmente, en un estudio realizado en Tailandia con 4.340 procedimientos quirúrgicos entre marzo y agosto de 2009; se encontró que durante la fase de trasferencia un no cumplimiento del 19.4% para el ítems del marcaje de los sitios quirúrgicos; durante la fase previa a la anestesia solo el 21% confirmó que todos los miembros del equipo se presentaron por nombre y rol, y solo un 21% de los eventos cruciales anticipados por el anestesista y eventos cruciales anticipados por el cirujano; durante la fase de previa al traslado a URPA el ítem de colección de espécimen quirúrgico con un 42.7% y confirmación oral de las preocupaciones clave para la recuperación y la gestión con un 14.9% (19).

Así mismo, en una investigación realizada en España, cuyo propósito fue llevar a cabo una intervención colaborativa con múltiples componentes y evaluar su impacto sobre la aplicación de la lista de chequeo de la cirugía propuesta por la OMS a través de un estudio multicéntrico longitudinal divido en tres etapas, en el cual participaron 27 hospitales. En dicho estudio se encontró que el porcentaje de cumplimiento global durante el estudio fue del 48%, siendo del 75,1% para el inicio de la etapa, 77,1% para la etapa intermedio y 88,3% para la etapa de cierre. El porcentaje de cumplimiento de cada ítem de la lista se mantuvo por arriba del 85% y la lista se chequeo se logró implementar con éxito en el 48% de las cirugías llevadas a cabo en los hospitales que participaron (20).

Estudios similares en el área han encontrado resultados similares de no cumplimiento, por ejemplo en un estudio llevado a cabo en India con 300 listas pertenecientes a pacientes pediátricos, se encontró que el 1,8% de los niños tenían los mismos nombres y procedimientos quirúrgicos idénticos publicados en la misma lista de operaciones, la manilla de identificación del paciente (0,1%), mención del lado de los procedimientos (3.6%), en el 0.1% de los pacientes se confundió la mención del lado de la operación en los documentos y los formularios de consentimiento, en el 2,6% de los pacientes, el formulario de consentimiento no fue firmado por los padres/tutores o el lado del procedimiento no fue marcado, los pedidos de antibióticos faltaban en 0,2% de los pacientes y en el 0,4% de casos, la inmovilización de los pacientes fue subóptima (17).

En relación con el cumplimiento por profesional, en este estudio se encontró diferencias entre los profesionales del equipo quirúrgico, siendo el personal de instrumentación quirúrgica el que presenta mayores niveles y el personal de enfermería el nivel más bajo. Este hecho es coincidente con el estudio de Ramírez y col (21), en el cual utilizaron listas de chequeo en cirugías programadas que mostraron un cumplimiento del 75.5% de la lista en el quirófano y se encontró una diferencia estadísticamente significativa (p<0,001) para las especialidades de cirugía general y ortopedia, así como entre el personal auxiliar de enfermería y el residente del equipo quirúrgico (p<0,001).

Con respecto al cumplimiento durante las fases de la cirugía en este estudio se encontró que el momento el momento de la cirugía previo a la anestesia alcanzó un 55.6%, seguido del momento previo a URPA que registro un cumplimiento del 37.78% y finalmente la fase de transferencia con un 24.4%. Similar al estudio anterior en una investigación realizada en Irán en donde evaluaron 1.771 cirugías; encontraron niveles de cumplimientos similares para cada una de las fases: 58% para la fase de transferencia, 16% durante la fase previa a la anestesia y 26% el momento previo a URPA (22).

En su estudio realizado en Argentina cuyo propósito fue evaluar los beneficios de la lista verificación para detección del origen de los errores cometidos en cirugías a través de un estudio observacional retrospectivo, en el cual se incluyeron 3.680 procedimientos quirúrgicos programados y auditaron el cumplimiento de la lista en los momentos pre, intra y postoperatorio inmediato. Durante el periodo de auditoria se presentaron 2.116 fallas (57,5%), de las cuales el 98,12% fueron atribuibles al factor humano y el 0,18% a factores técnicos-mecánicos. Adicionalmente por fases encontraron que durante el preoperatorio la falla más frecuente fue la ausencia de firma del consentimiento informado; en el intraoperatorio, la falta de previsión de eventos críticos, mientras que en el período postoperatorio inmediato fue la falta de protocolos operatorios y discrepancia en el recuento de gasas (23).

La implementación de los 19 ítems de la lista de chequeo propuesto por la OMS está asociado a aumento de la seguridad del acto quirúrgico. En un estudio cualitativo realizado en Brasil en el cual se aplicó la lista en 30 cirugías y a los miembros del equipo quirúrgico se le pregunto qué pensaban acerca de la aplicación de la lista, se pudo concluir que los sujetos no perciben que su uso le hubiera brindado más seguridad al procedimiento (24). Así mismo, Yuan y col (25), llevaron a cabo un estudio en dos hospitales de Liberia con recursos limitados, en los que se evaluaron 232 cirugías inicialmente, luego se entrenó al personal e implemento la lista de cheque de la OMS, posteriormente se evaluó 249 listas de chequeo y se encontró que la introducción de la lista de verificación se asoció con mejoras significativas en términos de procesos quirúrgicos generales y resultados quirúrgicos, así como en las mejoras en el cumplimiento a la seguridad.

Adicionalmente, en una revisión sistemática que incluyo 9 cohortes con estudios de controles históricos de cuatro entornos de atención hospitalaria: unidad de cuidados intensivos, departamento de urgencias, cirugía y cuidados agudos, los autores concluyeron que los artículos incluidos sugieren algunos beneficios del uso de listas de verificación de seguridad para mejorar el cumplimiento del protocolo y la seguridad del paciente (26). También, es un estudio llevado a cabo en pacientes de ortopedia en el cual se midió uso de la lista de verificación en pacientes ortopédicos antes y después de la implementación de un programa educativo; se recolectaron 480 pacientes antes del programa y 485 pacientes después, y se halló que el uso de la lista de verificación aumentó significativamente hasta 96.9% y además disminuyó la tasa de complicaciones y muerte (27).

Así mismo, Collazos y colaboradores (28) realizaron un estudio con el propósito de describir el proceso de implementación de la lista y evaluar el impacto sobre la reducción de los eventos adversos a través de la realización de estudio descriptivo en donde incluyeron 246 pacientes que recibieron cirugía mayor y se les pregunto acerca del cumplimento de los diferentes ítems durante al acto quirúrgico. En este estudio los pacientes refirieron que el ítem de menor cumplimiento fue la presentación de los miembros del equipo quirúrgico incluidos las funciones que llevaban a cabo con 85.8%, mientras que cuando se despertó después de la cirugía los pacientes refirieron que el solo el 40,7% del profesional de enfermería le dio recomendaciones para su cuidado después de la cirugía, así mismo el 96,8% de los pacientes refirió que recomendaría a otras personas que se operen en este hospital. Por otro lado, se observó una reducción alrededor del 4% de los eventos adversos luego de implementada la lista (7,26% en 2009 vs. 3,29% en 2010).

Por otra parte, en este estudio llama la atención que el personal de enfermería fue el que presentó el nivel más baja de cumplimiento por parte del equipo, situación que podría ser explicada dado que dicho personal es el encargado de liderar de manera trasversal la verificación y el correcto diligenciamiento de lista, pero en el momento de ejecutar sus actividades a cargo, las realiza de manera correcta, pero no tiene tiempo para dejar el respectivo registro en la misma, trayendo esto como consecuencia el no cumplimiento de todo el proceso, dado que el criterio empleado de cumplimiento en el presente estudio consistió no solo en realizar la actividad de manera correcta sino que además dejar el registro en la lista.

Por otra parte, una de las principales limitaciones de este estudio la constituye el efecto Hawthorne. Inicialmente el diligenciamiento de la lista de verificación de seguridad de la cirugía era incompleto, el personal quirúrgico hacia el llenado de la lista de manera anticipada a la fase siguiente sin haber llegado a esta, sin embargo, al sentirse observados en su labor en el quirófano el personal de salud empezó hacer el diligenciamiento de forma correcta sin errar, por lo cual el cumplimiento real podría estar sobreestimada. Sin embargo, dado que este estudio se tomó un criterio integral de cumplimiento de los ítems de la lista de seguridad quirúrgica propuesto por la OMS, esto llevo a que el nivel de cumplimiento registrado fuera muy bajo.

## Conclusiones

El cumplimiento general del equipo a la lista de chequeo fue muy bajo, lo cual comprueba la hipótesis de investigación, dado que el nivel de la lista resulto ser inferior al formulado inicialmente (<60% cumplimiento), siendo el ítem de profilaxis antibiótica el de menor cumplimiento, mientras que la fase previa a la anestesia la de mayor nivel. Sin embargo, dicho comportamiento es diferencial entre los miembros del equipo al ser las instrumentadoras quirúrgicas las que presentaron mayor cumplimiento y el personal de enfermería el menor.
